# *DSP* p.(Thr2104Glnfs*12) variant presents variably with early onset severe arrhythmias and left ventricular cardiomyopathy

**DOI:** 10.1186/s12881-020-0955-z

**Published:** 2020-01-31

**Authors:** Krista Heliö, Tiia Kangas-Kontio, Sini Weckström, Sari U. M. Vanninen, Katriina Aalto-Setälä, Tero-Pekka Alastalo, Samuel Myllykangas, Tiina M. Heliö, Juha W. Koskenvuo

**Affiliations:** 1 Heart and Lung Center, Helsinki University Hospital, University of Helsinki, Helsinki, Finland; 2 Blueprint Genetics, Helsinki, Finland; 3 Heart Center, Tampere University Hospital, Tampere, Finland; 4 Faculty of Medicine and Life Sciences, University of Tampere, Tampere, Finland

**Keywords:** Cardiomyopathies, Dilated cardiomyopathy, Arrhythmogenic cardiomyopathy, desmoplakin, DSP, Mutation

## Abstract

**Background:**

Dilated cardiomyopathy (DCM) is a condition characterized by dilatation and systolic dysfunction of the left ventricle in the absence of severe coronary artery disease or abnormal loading conditions. Mutations in the titin (*TTN*) and lamin A/C (*LMNA*) genes are the two most significant contributors in familial DCM. Previously mutations in the desmoplakin (*DSP*) gene have been associated with arrhythmogenic right ventricular cardiomyopathy (ARVC) and more recently with DCM.

**Methods:**

We describe the cardiac phenotype related to a *DSP* mutation which was identified in ten unrelated Finnish index patients using next-generation sequencing. Sanger sequencing was used to verify the presence of this *DSP* variant in the probands’ relatives. Medical records were obtained, and clinical evaluation was performed.

**Results:**

We identified *DSP* c.6310delA, p.(Thr2104Glnfs*12) variant in 17 individuals of which 11 (65%) fulfilled the DCM diagnostic criteria. This pathogenic variant presented with left ventricular dilatation, dysfunction and major ventricular arrhythmias. Two patients showed late gadolinium enhancement (LGE) and myocardial edema on cardiac magnetic resonance imaging (MRI) that may suggest inflammatory process at myocardium.

**Conclusions:**

The patients diagnosed with DCM showed an arrhythmogenic phenotype as well as SCD at young age supporting the recently proposed concept of arrhythmogenic cardiomyopathy. This study also demonstrates relatively low penetrance of truncating *DSP* variant in the probands’ family members by the age of 40. Further studies are needed to elucidate the possible relations between myocardial inflammation and pathogenic *DSP* variants.

## Background

Dilated cardiomyopathy (DCM) is characterized by left ventricular systolic dysfunction and dilatation of left ventricle in the absence of severe coronary artery disease or abnormal loading conditions such as hypertension or valvular disease [1]. Diagnosis is traditionally made by cardiac imaging, usually echocardiography. DCM is typically an adult-onset disease, but disease onset may take place already in infancy [2]. There is a wide variability in phenotypic expression and severity as clinical presentation varies from asymptomatic to end-stage heart failure or even sudden cardiac death (SCD) [2]. The prevalence of DCM in the general population is still unknown and it has previously been estimated to be 1:2500–1:3000 [3, 4]. These figures, however, are based on old data and the prevalence is now estimated to be much higher [5].

Familial DCM is typically considered to be a monogenic disorder and most commonly autosomal dominant inheritance has been reported [1, 2, 6, 7]. However, X-linked, recessive and mitochondrial inheritance patterns have been observed [6]. As much as 30–50% of DCM is thought to be genetic or familial [6, 8]. Over 40 genes have been related to DCM [8] and the most common genetic cause is a truncating variant in titin gene *(TTN)* [6, 9–11]. Mutations of lamin A/C gene (*LMNA*) are the second most common cause of DCM, causing 5–8% of familial DCM [12]. The most common causative genes in order of prevalence are *TTN, LMNA, DSP, MYH7* (beta-myosin heavy chain)*, RBM20* (RNA binding motif protein 20)*, TNNT2* (Troponin 2)*, TPM1* (tropomyosin 1)*, FLNC* (filamin C) and *DES* (desmin) [13, 14]*.* Mutations in sarcomeric proteins have some overlap to HCM [6]. Various genes coding cytoskeleton proteins such as *DSC2* (desmocollin-2) and *DSG2* (desmoglein 2) have also been associated with DCM and arrhythmogenic right ventricular cardiomyopathy (ARVC) [6].

*DSP* codes for the protein desmoplakin, which binds intermediate filament proteins to desmosomal plaques and is thus an essential part of a functional desmosome [15, 16]. Desmosomes are intercellular junctions that provide mechanical strength to tissues undergoing physical stress [15].

Earlier mutations in the *DSP* gene have mostly been associated with ARVC, which is an inherited cardiac disorder affecting usually the right ventricle [17–19]. The disorder is characterized by fibrofatty replacement, abnormal contraction and dilatation of the right ventricle (RV) [17, 20]. Ventricular arrhythmias often occur and may even lead to sudden cardiac death, especially in young people and athletes [20]. The diagnosis is based on the rather complex revised 2010 Task Force Criteria, and is a combination of major and minor criteria from different areas including RV function and structure, electrocardiogram (ECG) findings as well as genetic or familial background [21].

Previously the classification of cardiomyopathies has been based mainly on cardiac imaging or complex criteria, as for ARVC. More recently it has been observed that some phenotypes do not fit these earlier classifications. The concept of arrhythmogenic cardiomyopathy (ACM) has also been proposed, however, there are no diagnostic criteria for it so far. ACM comprises a group of cardiomyopathies with ventricular arrhythmias and right and/or left ventricular affection.

We found this *DSP* c.6310delA, p.(Thr2104Glnfs*12) variant interesting because it was found in a previous study in six patients who were diagnosed with DCM [13]. In this study we describe the cardiac phenotype related to this *DSP* variant in ten Finnish index patients and their family members.

## Methods

### Subjects

The study included ten index patients with the *DSP* variant c.6310delA, p.(Thr2104Glnfs*12), of which six participated earlier Finn-DCM study [13] and were recruited from Helsinki University Hospital and four were recruited from Tampere University Hospital. To better understand the manifestation of this *DSP* variant in the families, all the available family members were examined. All the participants gave written informed consent, and the study was approved by the Ethical Review Committee of The Department of Medicine, University of Helsinki (HYKS 26/99, HYKS 16/99, HYKS 17/99, HYKS 19/2000, HYKS 8/2000, Dnro 307/13/03/01/2011, TMK11§274,16.12.2015). We have the permission from Statistics Finland and Ministry of Social Affairs and Health to obtain clinical data from deceased patients for research purposes.

The probands with DCM were diagnosed with the following criteria: left ventricular end-diastolic diameter (LVEDD) > 27 mm/m^2^ and left ventricular systolic dysfunction (Left ventricular ejection fraction < 45%) in the absence of significant coronary artery disease or abnormal loading conditions such as hypertension or valvular disease. The relatives were also considered to be possibly affected if they had one or more of the following clinical abnormalities: conduction defects, atrial fibrillation (at age of < 50 years), sinus node dysfunction and dilatation or impaired systolic function of the left ventricle.

Family history was obtained, and pedigrees were drawn. All the available hospital records of the participants were acquired, and clinical data were collected from these records. Some of the patients were evaluated as part of this study by physical examination, 12-lead ECG, echocardiography and appropriate laboratory tests at the Heart and Lung Center in Helsinki. Cardiac magnetic resonance imaging (MRI), Holter, angiography and myocardial biopsy were performed in some cases. All participants of this study are of Finnish ethnicity.

### Molecular genetic studies

The genetic testing was carried out at the Blueprint Genetics laboratory at Helsinki, Finland. Eight index patients were tested using the Blueprint Genetics Pan Cardiomyopathy Panel covering 101 genes associated with cardiomyopathies and other large NGS panels including 72 and 133 genes were each used for one patient. The presence of the *DSP* variant in the probands’ family members was studied by using bi-directional Sanger sequencing.

Mutation nomenclature is based on GenBank accession NM_004415.2 (*DSP*) with nucleotide one being the first nucleotide of the translation initiation codon ATG. The pathogenicity of this *DSP* variant was evaluated based on the American College of Medical Genetics and Genomics (ACMG) classification scheme [22].

## Results

### Genetic studies

**Fig. 1:**
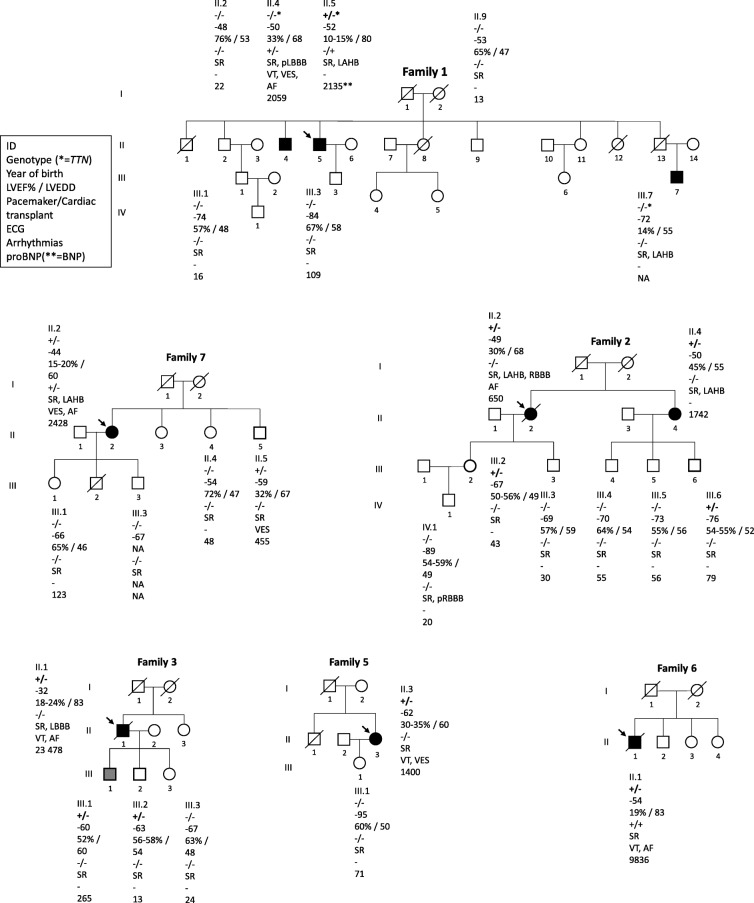
Pedigrees of six families affected with the *DSP* c.6310delA, p.(Thr2104Glnfs*12) variant. Squares represent men and circles women. Black-filled symbols represent individuals who fulfill DCM diagnostic criteria. Grey symbol represents individual who was considered affected. Arrows indicate index patients. Carriers of the *DSP* p.(Thr2104Glnfs*12) variant are marked in symbols with bolded outlines. Genotypes: +/− heterozygous for the *DSP* p.(Thr2104Glnfs*12), −/− wild type allele, * *TTN* p.(Val33411Thrfs*32). Year of birth, left ventricular ejection fraction and end-diastolic diameter and other clinical features listed below the symbols. LVEF left ventricular ejection fraction (%); LVEDD left ventricular end-diastolic diameter (mm). Pacemaker/Cardiac transplant: + indicates yes, − indicates no. ECG (electrocardiogram) - SR indicates sinus rhythm; (p)L/RBBB (partial) left/right bundle branch block; LAHB left anterior hemiblock. Arrhythmias - VT for ventricular tachycardia; VES for ventricular extrasystoles; AF for atrial fibrillation; NA not available. ProBNP pro b-type natriuretic peptide, ** BNP b-type natriuretic peptide

A heterozygous *DSP* c.6310delA, p.(Thr2104Glnfs*12) variant was observed in ten Finnish probands with cardiomyopathy. This variant causes a frameshift leading to a premature stop codon at residue 12 in a new reading frame. Thus, it is predicted to cause loss of normal protein function either through protein truncation (2114 out of 2871 aa) or nonsense-mediated mRNA decay from the other allele. It affects both RefSeq transcripts of DSP​​​​​​. There are 13 individuals heterozygous for this variant in the Genome Aggregation Database (gnomAD, *n* > 120,000 exomes and > 15,000 genomes). Of the heterozygotes, 11 are from the Finnish European cohort. Database curators have made every effort to exclude individuals with severe pediatric diseases from these cohorts. Altogether the variant was detected in 17 individuals of which 11 (65%) fulfilled DCM diagnostic criteria and 1/17 (6%) had a slightly dilated ventricle (118.8% of the estimated LVEDD using the Henry’s formula [23]). In two families the variant was observed only in the proband (Families 1 and 5, Fig. 1) and in four families (Families 6, 8, 9 and 11, Table 1) only the proband participated in the study. Pedigrees of the families (1-3 and 5-7) are shown in Fig. 1.

**Table 1:** The main clinical features of the index patients and their relatives

Family	Age (M/F)	Genotype, * = TTN+	Conduction defect	Arrhythmias	PM, ICD	LVEDD (mm)	EF (%)	proBNP (ng/l), ** = BNP	Age at dg	Phenotype	Other
**Family 1**											
II.2	68 M	−/−	AVB1	no	no	53	76	22	–		
II.4	65 M	−/−*	pLBBB	VT, VESm/R, AF	ICD	68	33	2059	58	DCM	
**II.5**	51 M	+/− *	LAHB	no	no	80	10–15	2135**	51	DCM	Heart transplant
II.9	63 M	−/−	no	no	no	47	65	13	–		
III.1	41 M	−/−	no	no	no	48	57	16	–		
III.3	31 M	−/−	no	no	no	58	67	109	–		
III.7	44 M	−/−*	LAHB	no	no	55	14	NA	44	DCM	
**Family 2**											
**II.2**	67F	+/−	LAHB, RBBB	AF	no	68	30	650	46	DCM	SCD
II.4	66F	+/−	AVB1, LAHB	no	no	55	45	1742	60	DCM	
III.2	49F	+/−	no	no	no	49	50–56	43	–		
III.3	47 M	−/−	no	no	no	59	57	30	–		
III.4	46 M	−/−	no	no	no	54	64	55	–		
III.5	42 M	−/−	no	bradycardia	no	56	55	56	–		
III.6	40 M	+/−	no	no	no	52	54–55	79	–		
IV.1	26 M	−/−	pRBBB	no	no	49	54–59	20	–		
**Family 3**											
**II.1**	81 M	+/−	AVB1, LBBB	VTm, AF	no	83	18–24	23,478	72	DCM	
III.1	56 M	+/−	no	no	no	60	52	265	–	Slightly dilated ventricle (Henry’s formula: 118.8%)	
III.2	53 M	+/−	no	no	no	54	56–58	13	–		
III.3	49F	−/−	no	no	no	48	63	24	–		
**Family 5**											
**II.3**	54F	+/−	AVB1, LAHB	VTm, VESp	no	60	30–35	1400	43	DCM	
III.1	21F	−/−	no	bradycardia	no	50	60	71	–		
**Family 6**											
**II.1**	59 M	+/−	AVB3	VT, AF	PM	83	19	9836	48	DCM	Heart transplant
**Family 7**											
**II.2**	70F	+/−	AVB2, LAHB	VESp, AF	CRT-D	60	15–20	2428	50	DCM	
II.4	62F	−/−	no	no	no	47	72	48	–		
II.5	57 M	+/−	no	VES	no	67	32	455	NA	DCM	
III.1	50F	−/−	no	no	no	46	65	123	–		
III.3	47 M	−/−	NA	NA	no	NA	NA	NA	–		
**Family 8**											
	48F	+/−	AVB1	VT	PM	70	22	3117	42	DCM	
**Family 9**											
	27F	+/−	no	VES	no	60	40	400	22	DCM	
**Family 10**											
II.1	M	+/−	no	VES	no	54	52	21	–		
**III.1**	14 M	+/−	no	VF	no	55	17	NA	14	DCM	SCD
**Family 11**											
	43 M	+/−	AVB2	no	no	52	52	20**	–		

Index patients are marked in bold. Symbols and abbreviations: Age (M/F) age and gender (M: male, F: female); genotype +/− heterozygous for the *DSP* p.(Thr2104Glnfs*12), −/− wild type allele, * *TTN* p.(Val33411Thrfs*32); *NA* not available; *AVB1–3* atrioventricular blocks type 1–3, (p)L/RBBB (partial)left/right bundle branch block; *LAHB* left anterior hemiblock; Arrhythmias - VT for ventricular tachycardia: VT means VT episode without information on QRS-axis or morphology, VT_m_ monomorphic VT and VT_p_ polymorphic VT; VES for ventricular extrasystoles: VES, when no information on VES morphology is available, VES_m/R_ means that most of the VES were monomorphic with RV origin (LBBB-morphology) and VES_p_ when most of the VES were polymorphic; *AF* for atrial fibrillation; VF for ventricular fibrillation; *PM* pacemaker; *ICD* implantable cardioverter-defibrillator; *CRT-D* cardiac resynchronization therapy device; *LVEDD* left ventricular end-diastolic diameter (mm); *LVEF* left ventricular ejection fraction (%); *ProBNP* pro b-type natriuretic peptide, ** BNP b-type natriuretic peptide; Age at dg - age at diagnosis of cardiomyopathy; Phenotype - phenotype at diagnosis; *DCM* dilated cardiomyopathy; Other – other significant clinical features; *SCD* sudden cardiac death

The main clinical features of the index patients and their relatives are presented in Table 1. These features include left ventricle end-diastolic diameter and ejection fraction, arrhythmias and conduction defects. Pacemaker or implantable cardioverter-defibrillator (ICD) was implanted in 3/17 (18%) of the heterozygotes and atrial fibrillation was observed in 4/17 (24%) and ventricular tachycardia or ventricular fibrillation in 5/17 (29%) of the cases. Four out of five DCM patients with severe arrhythmia episode (VT/VF) had cardiomyopathy changes prior or when first time presenting with arrhythmia episode. From one patient this information was not available. Conduction abnormalities were present in all but two of the families and atrioventricular-block (AV-block) of some degree was observed in 7/17 (41%) of the heterozygotes. Two out of 17 (12%) carriers of the *DSP* mutation had a heart transplantation and the other one also carried a heterozygous pathogenic *TTN* variant.

Nine patients underwent cardiac MRI and seven of them were carriers of the *DSP* variant. Of these seven, three fulfilled DCM diagnostic criteria, two had LGE as well as cardiac edema, one had only LGE. Fibrofatty replacement was not identified in any of the patients.

Two out of seven genotype positive family members had DCM at ages 57 and 60, one had dilated LV with normal EF at age 56, one had second degree AV block at age 43 and three others were considered as genotype positive yet phenotype negative at ages 40, 49 and 53. All genotype negative family members were unaffected.

Based on the variant classification scheme that follows the ACMG guidelines, this *DSP* c.6310delA, p.(Thr2104Glnfs*12) variant can be considered as pathogenic.

### Family 1

Proband (II.5) of this family was diagnosed with DCM at the age of 51. His BNP (b-type natriuretic peptide) concentration was 2135 ng/l at its highest. He had no history of arrhythmias, but ECG showed left anterior hemiblock. On echocardiography his LVEDD was 80 mm and LVEF 10–15%. The proband later received a heart transplant. Myocardial biopsy was taken, and no amyloidosis was observed. In addition to the *DSP* variant, the proband also had a pathogenic variant, *TTN* c.100230_100234delinsGACA, p.(Val33411Thrfs*32); NM_001267550.1. The same *TTN* deletion, but not the *DSP* variant, was also found in two other members of the family, the proband’s brother (II.4) and nephew (III.7) who both were affected.

### Family 2

Proband (II.2) of this family was a 67-year-old female diagnosed with DCM at the age of 46. The patient had suffered from atrial fibrillation and ECG showed right bundle branch block as well as left anterior hemiblock. Her pro-BNP (pro b-type natriuretic peptide) concentration was 650 ng/l and on echocardiography her LVEDD was 68 mm and LVEF 35%. The proband died at the age of 69 suddenly. In autopsy both cardiac ventricles were enlarged, there was no significant coronary artery disease, neither signs of myocardial infarct nor pulmonary embolism. The cause of death was considered to be cardiac. Proband’s genotype positive sister (II.4) was diagnosed with DCM at the age of 60. She has no arrhythmias, but ECG showed 1-degree AV block and left anterior hemiblock. On echocardiography her LVEDD was 55 mm and LVEF 45%. Her pro-BNP concentration was 1742 ng/l at its highest. Proband’s 49-year-old genotype positive daughter (III.2) had normal findings in cardiac evaluations. At first cardiac evaluation proband’s genotype positive 40-year-old nephew (III.6) had normal findings. One year later a cardiac MRI was performed and his LVEDV (Left Ventricular End-Diastolic Volume) was 182 ml, LVEF 54%, RVEDV (Right Ventricular End-Diastolic Volume) 173 ml and RVEF (right ventricular ejection fraction) 56%. There was considerable acute edema in the basal and mid-third anterolateral left ventricle as well as subepicardial intensive late gadolinium enhancement (LGE) within the same area.

### Family 3

Proband (II.1) is an 81-year-old male diagnosed with DCM at the age of 72. He has had ventricular tachycardia as well as atrial fibrillation. ECG showed a first-degree AV block and left bundle branch block. The proband had very high levels of pro-BNP (23,478 ng/l) and on echocardiography his LVEDD was 83 mm and LVEF 18–24%. Proband’s both sons (III.1, III.2) were found to be heterozygous for the *DSP* variant. Proband’s eldest son (III.1) is a 56-year-old male with coronary artery disease. He has no conduction defects nor arrhythmias. On echocardiography his LVEDD was 60 mm (118.8% of the estimated LVEDD using the Henry’s formula) and LVEF 52%. Cardiac MRI demonstrated a slightly dilated left ventricle with impaired function and the wall-thickness in inferolateral area was 3–4 mm locally. Proband’s other son (III.2) is a 53-year-old male who had normal findings in cardiac evaluations.

### Family 5

Proband (II.3) was diagnosed with DCM at the age of 43. She suffered from ventricular tachycardia and ventricular extrasystoles. On echocardiography her LVEDD was 60 mm and LVEF 30–35%, ECG showed a 1-degree AV block and left anterior hemiblock. Cardiac MRI demonstrated a dilated left ventricle with end-diastolic volume of 219 ml and LVEF of 41%. Her pro-BNP concentration was 1400 ng/l at its highest.

### Family 6

Proband (II.1) was diagnosed with DCM at the age of 48. He had high levels on pro-BNP (9836 ng/l) and on echocardiography his LVEDD was 83 mm and LVEF 19%. He had mild mitral regurgitation and later developed a 3-degree AV block. He had ventricular tachycardia as well as atrial fibrillation and he later received a pacemaker. At the age of 59 he received a heart transplant. The proband was the only member of this family to participate in the study.

### Family 7

Proband (II.2) is a 70-year-old female who developed DCM by the age of 50 years. ECG showed 2-degree AV block and left anterior hemiblock. She suffers from ventricular extrasystoles and atrial fibrillation. She has later received a CRT-D pacemaker. On echocardiography her LVEDD was 60 mm and LVEF 15–20%. Her pro-BNP concentration was 2428 ng/l at its highest. Proband’s genotype positive brother (II.5) is a 57-year-old male. ECG showed no conduction defects, but he has had ventricular extrasystoles. On echocardiography his LVEDD was 67 mm and LVEF 32%. His pro-BNP concentration was 455 ng/l. Proband’s son (III.2) died in car accident.

### Family 8

Proband is a 48-year-old female, diagnosed with DCM at the age of 42. She has suffered from ventricular tachycardia and ECG showed a 1-degree AV block. She has later received a pacemaker. Echocardiography showed mild mitral regurgitation and her LVEF was 22% and LVEDD 70 mm. Cardiac MRI demonstrated a dilated left ventricle, LVEF of 20%, reduced wall-thickness and no fibrosis. Her pro-BNP was 3117 ng/l at its highest. The proband was the only member of the family to participate in the study.

### Family 9

Proband is a 27-year-old female who was diagnosed with DCM at the age of 22. ECG showed no conduction defects, but she has suffered from ventricular extrasystoles. On echocardiography her LVEDD was 60 mm and LVEF 40%. At its highest her pro-BNP concentration was 400 ng/l. Cardiac MRI demonstrated an increase in signal intensity on the lateral wall of the left ventricle and there was also patchy LGE on the lateral left ventricular wall subepicardially and on the right ventricle side of the septum. Also, slightly elevated Troponin T (TnT) concentrations were observed (TnT 18–39 ng/l, normal range < 14 ng/l). Endomyocardial biopsy was taken 6 months later and it showed variation in nucleolar shape and size and myodegeneration but no sign of myocarditis. The proband was the only member of this family to participate in the study.

### Family 10

Proband (III.1) was an apparently healthy 14-year-old male, who had ventricular fibrillation while watching TV at home. He was resuscitated but succumbed 2 days later at the hospital. At the hospital ECG showed no conduction defects, and on echocardiography his LVEF was 17% and LVEDD 55 mm. No known Long QT Syndrome (LQTS) causing variants were found on genetic evaluation. On autopsy, histology did not demonstrate findings compatible with acute myocarditis. There were no signs of amyloidosis or hemochromatosis. Plakoglobin staining was normal. Interstitial fibrosis of varying grades could be observed. Proband’s genotype positive father (II.1) has had ventricular extrasystoles whereas ECG showed no conduction defects. His pro-BNP concentration was 21 ng/l and on echocardiography his LVEDD was 54 mm (111% of the estimated LVEDD using the Henry’s formula) and LVEF 52%. Cardiac MRI demonstrated no clear LGE.

### Family 11

Proband is a 43-year-old male with a 2-degree AV-block. Cardiac MRI demonstrated slightly dilated ventricles and atriums, LVEF was 58% and RVEF 60%. There was no edema. Linear and midmyocardial LGE compatible with fibrosis was observed. Proband was the only member of the family that could be recruited to this study and we have no knowledge of proband’s parents’ genotypes, but we do know the following considering their cause of death. Proband’s mother died suddenly at the age of 24 while pulling a sled on the street. During the 4 years that preceded her death, she had suffered from palpitations of unknown cause and she had visited the hospital several times because of chest pains. On the last of these visits, a 1-degree AV-block was observed. Proband’s father had alcoholic liver cirrhosis and he died suddenly at later age due to intoxication.

## Discussion

We have identified the *DSP* c.6310delA, p.(Thr2104Glnfs*12) in ten Finnish index patients with DCM. This study also demonstrates relatively low penetrance of truncating *DSP* variant in the probands’ family members before age of 40 but phenotype became evident in about half of the family members by age 60. In addition to the previous FinnDCM study [13], this variant has previously been reported twice in literature, however, in both cases the patients were also carriers of another *DSP* gene mutation. In both cases the phenotype was cardio-cutaneous and in the other case, the patient suffered sudden cardiac death [24, 25].

Nine of the index patients and two relatives fulfilled the diagnostic criteria for DCM but at least one of the subjects, a young previously healthy individual, had ventricular fibrillation and SCD as the first manifestation of the disease. The age of diagnosis varied greatly as the youngest was 14 years old while the oldest participant was 72 years old when the diagnosis was made. Two of the participants presented edema on cardiac MRI. From one of these patients TnT was measured and initially elevated, then normalized in follow-up. The findings were suggestive of some kind of inflammatory process. The inflammatory process in the myocardium could explain some of the sudden arrhythmias as the disease might process periodically rather than continuously. However further studies are needed to elucidate the possible relations between *DSP* pathogenic variants and myocardial inflammation.

The major findings in our subjects are ventricular arrhythmias and dilatation of the left ventricle. Three of the patients died suddenly and two of them at young age. Our findings confirm the significance of *DSP* gene as a cause of arrhythmogenic cardiomyopathy.

Even though some of the patients do meet the DCM diagnostic criteria, it seems that the arrhythmogenic side of this phenotype is more important in practice, because the deaths were caused by arrhythmogenic events rather than ventricular dysfunction.

Desmoplakin is coded by the *DSP* gene on chromosome 6p24.3, and there are two isoforms produced by alternative splicing. Desmoplakin is a crucial part of forming functional desmosomes as it interacts with intermediate filaments and binds them to desmosomal plaques [15, 16]. Desmosomes are intercellular junctions that are abundant in tissues undergoing constant physical stress, such as the heart and the epidermis [15]. To adapt to their mechanical environment, cardiomyocytes may use different proteins such as integrins or strain-activated ion channels [26]. The mechanical stresses the cell experiences can be transmitted through the cytoskeleton to the nucleus by actin, intermediate filaments and microtubules [26]. The main intermediate filaments in cardiac myocytes are desmins which are connected to desmosomes. A recent study has suggested that the junction between intermediate filaments and desmosomes is specialized to endure external mechanical stress and having a stress absorbing function [27]. Desmoplakin carries a significant role in this as under most conditions it does not experience significant tension, but it is capable of sensing the exposure to external mechanical stresses and reacting to it [27].

Mutations in *DSP* manifest in the skin, hair and heart in humans. The participants in this study were not studied systematically for skin or hair abnormalities, but there were no mentions of these kinds of defects in the patient records. Autosomal recessively inherited mutations have previously been associated with severe disorders such as Carvajal syndrome [28–30], skin fragility-woolly hair syndrome and lethal acantholytic epidermolysis bullosa [31]. Autosomal dominant mutations in *DSP* have been associated with ARVC [32], DCM and SAM (Severe skin dermatitis, multiple allergies and metabolic wasting) syndrome [33]. There are altogether 163 truncating *DSP* variants reported in ClinVar (May 2019) of which 156 (95,7%) were classified as pathogenic or likely pathogenic. Most of the variants classified as non-pathogenic are located in the C-terminus.

According to gnomAD the *DSP* c.6310delA, p.(Thr2104Glnfs*12) frequency in Finnish population is 0.0005081. Total allele frequency is 0.00005170. The variant is enriched in Finnish population but the presence of this variant in the reference population could possibly be explained by the variant’s low penetrance before the age of 40. Because of the late expression of the phenotype, this could lead to the inclusion of variant carriers in the reference population, who have not yet developed the disease.

According to the 2008 position statement of the European Society of Cardiology, cardiomyopathies have been divided into groups based on the phenotypes and each phenotype is divided into familial and non-familial forms [1]. DCM is defined by dilatation and systolic dysfunction of the left ventricle when no abnormal loading conditions or coronary artery disease are observed [1]. Right ventricular dilatation and dysfunction may also be observed although it is not necessary for the diagnosis.

Arrhythmogenic right ventricular cardiomyopathy is a myocardial disorder defined by the dysfunction of the right ventricle and the left ventricle may also be affected. Histologically ARVC is characterized by fibrofatty replacement of the myocardium, abnormal contraction and dilatation of the right ventricle. The diagnosis is based on the latest revised Task Force diagnostic criteria, that was published in 2010 [21]. Mutations in desmosomal genes, such as *PKP2* (plakophilin 2)*, DSP, DSG2* and *DSC2,* have been associated with ARVC [18]. The 2008 Task Force ARVC diagnostic criteria does not take in to account the presence of left ventricular dysfunction. The 2010 criteria also include the biventricular and LV subtypes, but there are no specific diagnostic criteria for the non-classical disease patterns [21].

Not all of our participants had their right ventricles systematically evaluated, but no abnormalities in the size or function of the right ventricle on echocardiography were reported in hospital records. Those who had cardiac MRI performed, did not show any defects in the function nor structure of the right ventricles.

This is in line with some previous studies, in which it has been noted that *DSP* mutations seem to be associated with left dominant or biventricular form of ARVC rather than the classical right dominant form, and *DSP* mutation carriers have a higher risk of SCD when compared to other mutation carriers [19, 34–36]. Especially the truncating mutations of *DSP* were associated with higher risk of developing LV dysfunction in ARVC [19].

Increasing amount of reports of clinical variants characterized by left ventricular involvement which may be parallel or greater than the RV involvement, has led to the use of a broader term of “arrhythmogenic cardiomyopathy” (ACM) [20]. Arrhythmogenic cardiomyopathy is characterized by fibrofatty replacement of the myocardium and ventricular arrhythmias as well as impairment of ventricular function. While ARVC is focused on the impairment of the right ventricle, ACM is a broader term and also includes biventricular and left-dominant subtypes [20, 37]. Arrhythmogenic left ventricular cardiomyopathy (ALVC) refers to a phenotype of left ventricular cardiomyopathy with ventricular arrhythmias, however, no diagnostic criteria have been established. As the genetic background of arrhythmogenic cardiomyopathies is gradually revealed, it will also be possible to better characterize the related phenotypic variation.

## Conclusion

This s.6310delA *DSP* variant causes an arrhythmogenic phenotype in addition to the suboptimal function of the left ventricle although fibrofatty infiltration was not observed. Our findings concur with the proposed concept of arrhythmogenic cardiomyopathy but more research in the subject is still needed. The variant is more common in the Finnish population as compared to many other populations. It might also modify the risk of ventricular arrhythmias for example in the context of coincidental coronary artery disease. Understanding the genetic background may have some significance in the diagnosis and treatment of these diseases in the future.

## Data Availability

The datasets generated and/or analysed during the current study are not publicly available due to privacy issues and GDPR legislation but are available from the corresponding author on reasonable request.

## Abbreviations

ACM: Arrhythmogenic cardiomyopathy
ACMG: American College of Medical Genetics and Genomics
ALVC: Arrhythmogenic left ventricular cardiomyopathy
ARVC: Arrhythmogenic right ventricular cardiomyopathy
AV-block: Atrioventricular block
BNP: B-type natriuretic peptide
DCM: Dilated cardiomyopathy
DES: Desmin
DSC2: Desmocollin-2
DSG2: Desmoglein 2
DSP: Desmoplakin
ECG: Electrocardiogram
FLNC: Filamin C
ICD: Implantable cardioverter-defibrillator
LGE: Late gadolinium enhancement
LMNA: Lamin A/C
LQTS: Long QT Syndrome
LV: Left ventricle
LVEDD: Left ventricular end-diastolic diameter
LVEDV: Left Ventricular End-Diastolic Volume
LVEF: Left ventricular ejection fraction
MRI: Magnetic resonance imaging
MYH7: Beta-myosin heavy chain
PKP2: Plakophilin 2
pro-BNP: Pro b-type natriuretic peptide
RBM20: RNA binding motif protein 20
RVEDV: Right Ventricular End-Diastolic Volume
RVEF: Right ventricular ejection fraction
SAM: Severe skin dermatitis, multiple allergies and metabolic wasting
SCD: Sudden cardiac death
TNNT2: Troponin T2
TnT: Troponin T
TPM1: Tropomyosin 1
TTN: Titin
